# Atorvastatin Safety Reappraised: From Pharmacogenomic Mechanisms to Personalized Risk Management

**DOI:** 10.1155/cdr/4127131

**Published:** 2026-10-02

**Authors:** Hanyu Wang, Yue Pan, Yiming Li, Pan Zhai

**Affiliations:** ^1^ Department of Critical Care Medicine Zhongnan Hospital of Wuhan University Wuhan Hubei Province China znhospital.cn; ^2^ Department of Integrated Traditional Chinese Medicine and Western Medicine Zhongnan Hospital of Wuhan University Wuhan Hubei Province China znhospital.cn

**Keywords:** atorvastatin, drug–drug interactions, hepatotoxicity, myotoxicity, pharmacogenomics, pharmacokinetics, pharmacovigilance

## Abstract

Atorvastatin remains a cornerstone of lipid‐lowering therapy and cardiovascular prevention, but concerns about adverse effects continue to undermine adherence and long‐term clinical benefit. This structured narrative review critically reappraises its safety profile by integrating evidence from randomized trials, meta‐analyses, observational studies, pharmacovigilance analyses, case reports, and preclinical models. Evidence is interpreted according to study design, with controlled and synthesized human data guiding clinical conclusions and lower level evidence treated as supportive or hypothesis‐generating. Overall, atorvastatin provides substantial cardiovascular protection, whereas serious adverse reactions are uncommon. The principal clinical concerns are statin‐associated muscle symptoms, hepatic laboratory abnormalities, drug–drug interactions, and a small dose‐ and susceptibility‐dependent increase in new‐onset diabetes. Safety is shaped by dose, comorbidities, interacting medicines, pharmacokinetic variability, and transporter variants, particularly SLCO1B1, although the clinical actionability of other pharmacogenomic markers remains limited. We, therefore, emphasize individualized risk assessment, interaction review, appropriate monitoring, dechallenge and rechallenge, dose adjustment, alternative statins, and nonstatin combination therapy when needed. Experimental delivery systems and mechanistic findings may inform future strategies but do not yet establish improved clinical safety. Taken together, the evidence supports a benefit‐centered, evidence‐stratified framework that addresses genuine toxicity without overstating uncertain safety signals or unnecessarily discontinuing effective therapy.

## 1. Introduction

Atorvastatin is a synthetic member of the statin class and competitively inhibits 3‐hydroxy‐3‐methylglutaryl coenzyme A reductase, reducing hepatic cholesterol synthesis and upregulating low‐density lipoprotein receptors [1]. It is approved for primary hyperlipidemia, heterozygous and homozygous familial hypercholesterolemia, hypertriglyceridemia, and reduction of cardiovascular risk in appropriate adults and pediatric patients [2, 3]. Available tablet strengths are 10, 20, 40, and 80 mg; the usual adult starting dose is 10–20 mg once daily, with a 10–80 mg/day range [2, 4]. Atorvastatin is rapidly absorbed, has approximately 14% parent drug bioavailability because of presystemic clearance, and is metabolized mainly by CYP3A4 to active metabolites [1, 5]. These features support once‐daily dosing but also create clinically relevant interaction risks. Figure 1 summarizes the principal mechanism of atorvastatin, including inhibition of the mevalonate pathway, upregulation of hepatic LDL receptors, enhanced LDL‐C clearance, and the pathways underlying its pleiotropic effects. Against this pharmacological background, the present review focuses on safety while maintaining the established cardiovascular benefit as the clinical reference point.

**Figure 1:**
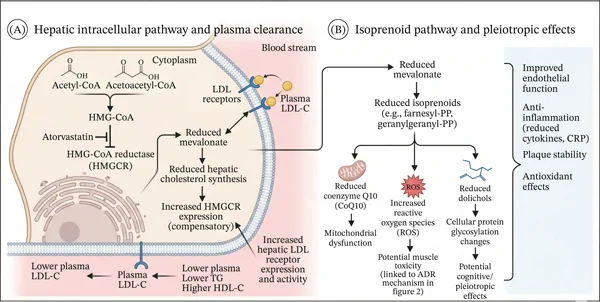
Refined mechanism of action of atorvastatin: cholesterol synthesis inhibition and pleiotropic effects. This figure illustrates the dual pharmacological pathways of atorvastatin. (A) Hepatic intracellular pathway and plasma clearance: Atorvastatin competitively inhibits HMG‐CoA reductase (HMGCR), the rate‐limiting enzyme in cholesterol synthesis, leading to reduced production of mevalonate. This process triggers a compensatory increase in HMGCR expression and the upregulation of hepatic low‐density lipoprotein receptors (LDLRs), which enhances the clearance of plasma LDL‐C, ultimately lowering total cholesterol and triglyceride levels. (B) Isoprenoid pathway and pleiotropic effects: The reduction in mevalonate also leads to the depletion of downstream isoprenoids, such as farnesyl pyrophosphate and geranylgeranyl pyrophosphate. This depletion results in two distinct outcomes: (1) pleiotropic cardiovascular benefits, including improved endothelial function, anti‐inflammatory effects (reduced cytokines and CRP), and plaque stability, and (2) potential adverse drug reaction (ADR) mechanisms, where reduced levels of coenzyme Q10 (CoQ10) and dolichols lead to mitochondrial dysfunction, increased reactive oxygen species (ROS), and altered protein glycosylation, forming the biochemical basis for muscle toxicity and neurocognitive side effects.

The core challenges in managing atorvastatin safety involve a complex interplay of pharmacological, genetic, and psychological factors. Statin‐associated muscle symptoms (SAMSs) represent the most frequent clinical hurdle, ranging from mild myalgia to rare but life‐threatening rhabdomyolysis [6–9]. Interestingly, research suggests a substantial “nocebo effect,” where symptoms reported by patients often do not differ significantly from placebo in blinded trials [10–12]. Beyond musculature, research has highlighted concerns regarding hepatotoxicity [13, 14], new‐onset Type 2 diabetes [1, 15, 16], and rare idiosyncratic reactions such as cognitive impairment [17], psychiatric disturbances [18], and even dermatological or immunological responses like angioedema and lupus‐like syndromes [19, 20]. Technical difficulties persist in distinguishing true pharmacological causation from coincidental symptoms, exacerbated by drug–drug interactions with agents like gemfibrozil or sacubitril/valsartan [21–23], and interindividual variability driven by genetic polymorphisms in transporters and enzymes such as SLCO1B1, ABCG2, and CYP3A4 [12, 24–26].

This review critically evaluates atorvastatin safety across musculoskeletal, hepatic, metabolic, renal, neuropsychiatric, immunologic, and other systems, compares atorvastatin with commonly prescribed statins, and examines pharmacokinetic, drug interaction, and pharmacogenomic determinants of risk. Clinical conclusions are based primarily on systematic reviews, meta‐analyses, and randomized trials. Observational studies provide complementary estimates of real‐world effectiveness and uncommon outcomes, whereas pharmacovigilance analyses, case reports, and preclinical experiments are interpreted as supportive or hypothesis‐generating evidence rather than proof of causality.

### 1.1. Literature Search and Evidence Appraisal

A structured narrative literature search was conducted in PubMed, supplemented by backward citation checking of relevant reviews and primary studies, from database inception through May 31, 2026. The primary PubMed search strategy was (“Atorvastatin”[MeSH Terms] OR atorvastatin[Title/Abstract]) AND (safety[Title/Abstract] OR adverse effect∗[Title/Abstract] OR adverse event∗[Title/Abstract] OR adverse drug reaction∗[Title/Abstract] OR tolerability[Title/Abstract] OR statin‐associated muscle symptom∗[Title/Abstract] OR muscle symptom∗[Title/Abstract] OR muscle pain[Title/Abstract] OR myalgia[Title/Abstract] OR myopathy[Title/Abstract] OR rhabdomyolysis[Title/Abstract] OR diabet∗[Title/Abstract] OR dysglyc∗[Title/Abstract] OR hyperglyc∗[Title/Abstract] OR blood glucose[Title/Abstract] OR glucose intolerance[Title/Abstract] OR glycem∗[Title/Abstract] OR glycaem∗[Title/Abstract] OR hepatotox∗[Title/Abstract] OR liver injur∗[Title/Abstract] OR hepatic dysfunction[Title/Abstract] OR liver dysfunction[Title/Abstract] OR hepatic impairment[Title/Abstract] OR liver impairment[Title/Abstract] OR liver failure[Title/Abstract] OR abnormal liver function[Title/Abstract] OR liver enzyme∗[Title/Abstract] OR aminotransferase∗[Title/Abstract] OR transaminase∗[Title/Abstract] OR pharmacokinetic∗[Title/Abstract] OR drug interaction∗[Title/Abstract] OR pharmacogenomic∗[Title/Abstract] OR SLCO1B1[Title/Abstract] OR ABCG2[Title/Abstract] OR ABCB1[Title/Abstract] OR UGT1A1[Title/Abstract] OR pharmacovigilance[Title/Abstract]) AND 1000/01/01:2026/05/31[Date ‐ Publication]. Supplementary statin class searches were conducted for outcomes not adequately captured by atorvastatin‐specific records, particularly SAMSs, dysglycemia, and new‐onset diabetes. Targeted combinations with randomized trial, meta‐analysis, observational study, case report, animal, and in vitro terms were used to identify evidence by study design. English‐language human studies reporting efficacy, safety, adverse‐event frequency, comparative outcomes, pharmacokinetics, drug interactions, or genotype‐related outcomes were prioritized. Animal and in vitro studies were included only when they addressed clinically relevant mechanistic hypotheses. Duplicate reports, noninformative commentaries, and studies without atorvastatin‐specific or relevant statin class safety information were excluded. Evidence was appraised qualitatively according to study design, sample size, comparator, outcome ascertainment, precision, consistency, and clinical applicability. Because no protocol was prospectively registered and study selection was not designed to meet PRISMA requirements, this article is described as a structured narrative review rather than a systematic review.

## 2. Clinical Safety, Tolerability, and Dose–Response of Atorvastatin

Randomized evidence establishes both efficacy and the scale of clinical benefit. Atorvastatin 10–80 mg/day lowers LDL‐C by approximately 37% and 52% [1, 4], and each 1.0 mmol/L reduction in LDL‐C is associated with an approximately 21% proportional reduction in major vascular events [27]. The main dose‐related clinical concerns are SAMS and aminotransferase elevation, whereas rhabdomyolysis is very uncommon [28–30]. In the Treating to New Targets (TNT) trial, atorvastatin 80 versus 10 mg/day reduced major cardiovascular events from 10.9% to 8.7% (hazard ratio 0.78, 95% confidence interval 0.69–0.89), but persistent aminotransferase elevations increased from 0.2% to 1.2% [31]. Across trials, overall tolerability is favorable [2, 32, 33]. Accordingly, dose selection should reflect baseline cardiovascular risk, the required LDL‐C reduction, interacting drugs, frailty, hepatic function, and prior intolerance rather than potency alone [5, 34–36].

### 2.1. Comparative Effectiveness of Atorvastatin and Other Statins

Comparisons among statins should distinguish lipid‐lowering potency from clinical safety and study design [37]. Atorvastatin and rosuvastatin are high‐intensity options at appropriate doses, whereas simvastatin, pravastatin, fluvastatin, and pitavastatin generally provide low‐ to moderate‐intensity therapy. Detailed dose intensity ranges are summarized in Table 1. However, observational head‐to‐head studies can be affected by prescribing differences and residual confounding, so claims of superior tolerance or mortality should not be generalized without qualification. Pharmacokinetic differences are clinically useful: Atorvastatin and simvastatin are susceptible to CYP3A4‐mediated interactions, whereas pravastatin, rosuvastatin, and pitavastatin have less CYP3A4 dependence [38]. Statin choice should, therefore, integrate the required LDL‐C reduction, comorbidities, interacting drugs, renal and hepatic function, prior SAMS, and cost [39]. Table 1 compares commonly prescribed statins in terms of LDL‐C‐lowering intensity, pharmacokinetics, interaction potential, clinical advantages, and major limitations.

**Table 1:** Comparative clinical characteristics of commonly prescribed statins.

Statin	Approximate LDL‐C lowering/intensity	Key pharmacokinetics and interactions	Clinical role, advantages, and limitations
Atorvastatin [3–5, 29]	10 mg: ~37%–40%; 40–80 mg: High intensity (~50% or more)	CYP3A4 substrate; OATP1B1‐mediated uptake; interaction risk with strong CYP3A4/OATP inhibitors	Broad cardiovascular indications; no renal dose adjustment solely for clearance; high‐dose SAMS and aminotransferase risk; SLCO1B1 guidance available
Rosuvastatin [16, 40, 41]	5–10 mg: Moderate; 20–40 mg: High intensity	Limited CYP3A4 metabolism; transporter‐mediated disposition; renal function affects exposure	High potency at lower milligram doses; fewer CYP3A4 interactions; dose constraints in severe renal impairment and some ancestry groups
Simvastatin [21, 42]	10–40 mg: Low to moderate intensity	CYP3A4 substrate; strong SLCO1B1 effect	Low cost and extensive outcome evidence; more interaction‐ and genotype‐sensitive; 80 mg generally avoided
Pravastatin [43–45]	10–80 mg: Low to moderate intensity	Minimal CYP metabolism; renal elimination contributes	Few CYP3A4 interactions; lower potency; renal dose considerations
Fluvastatin [46, 47]	20–80 mg: Low to moderate intensity	Predominantly CYP2C9	Lower interaction burden with CYP3A4 inhibitors; lower potency and CYP2C9‐related exposure variability
Pitavastatin [44, 48]	1–4 mg: Moderate intensity	Minimal CYP metabolism; transporter‐mediated disposition	Useful when CYP3A4 interactions are a concern; less outcome evidence and variable availability/cost

## 3. Atorvastatin‐Associated Adverse Effects and Organ Toxicity

Safety signals differ substantially by evidence source. Randomized controlled trials provide the most reliable comparison of common adverse events and causal effects but may exclude frail or highly comorbid patients and are often underpowered for rare outcomes. Meta‐analyses improve precision but inherit heterogeneity in adverse‐event definitions. Observational cohorts improve generalizability and can assess longer follow‐up, yet confounding by indication and differential discontinuation remain important. Spontaneous reporting databases and case reports are valuable for detecting unusual events but cannot estimate incidence or establish causality. Animal and in vitro studies clarify possible mechanisms but do not demonstrate clinical harm at therapeutic exposure. The following organ system sections, therefore, identify the evidence level explicitly and base clinical conclusions primarily on randomized and synthesized human evidence. Table 2 summarizes the major atorvastatin‐related adverse drug reactions by organ system, approximate frequency, clinical manifestations, and strength of supporting evidence.

**Table 2:** Summary of atorvastatin‐related adverse drug reactions (ADRs) by system and mechanism.

Affected system	Adverse reaction	Approximate frequency/evidence	Main manifestations	Interpretation/mechanism
Musculoskeletal	SAMS/myalgia [6, 7, 49, 8, 12]	~1%–3% in blinded trials; ~5%–10% in observational practice, with higher estimates in selected cohorts	Symmetrical pain, tenderness, stiffness, or weakness; CK often normal	Multifactorial; assess dose, interactions, thyroid disease, exertion, and expectation effects
Musculoskeletal	Myopathy/rhabdomyolysis [6–9, 21, 34, 35]	Myopathy uncommon; rhabdomyolysis generally < 0.1%	Marked CK elevation; myoglobinuria and acute kidney injury in severe cases	Risk increases with high dose, interacting drugs, frailty, renal impairment, and selected genotypes
Hepatic	Persistent aminotransferase elevation > 3x ULN [13–15, 30, 34, 35]	~0.5%–0.7% overall; 0.2% with 10 mg vs. 1.2% with 80 mg in TNT	Usually asymptomatic; clinically apparent liver injury is rare	Dose‐related laboratory effect; idiosyncratic injury requires exclusion of alternative causes
Metabolic	New‐onset diabetes [50, 51]	~9%–12% relative increase at class level; absolute risk depends on baseline glycemia and intensity	Progression across diagnostic glucose/HbA1c threshold	Monitor patients with obesity, impaired fasting glucose, or metabolic syndrome; cardiovascular benefit usually predominates
Neuropsychiatric	Cognitive/psychiatric symptoms [18, 17]	Frequency uncertain; mainly case reports and spontaneous reporting associations	Memory complaints, insomnia, nightmares, or mood symptoms	Causality not established; evaluate competing causes and temporal relationship
Renal/pancreatic/immune	AKI, pancreatitis, and hypersensitivity [19, 23, 36, 52–54]	Rare or frequency not estimable from spontaneous reports and cases	Organ‐specific symptoms; AKI may follow rhabdomyolysis	Signals require clinical verification; spontaneous reports lack denominators

*Note:* Frequencies vary according to study design, population, dose, adverse‐event definition, and ascertainment method.

Abbreviations: ADR, adverse drug reaction; CK, creatine kinase; SAMS, statin‐associated muscle symptom; ULN, upper limit of normal.

### 3.1. Skeletal Muscle Adverse Effects and SAMSs

SAMS ranges from myalgia with normal creatine kinase to uncommon myopathy and very rare rhabdomyolysis or immune‐mediated necrotizing myopathy [6, 8]. Reported frequency depends strongly on study design: Blinded randomized trials generally report SAMS in approximately 1%–3% of participants, whereas observational studies report about 5%–10%, with higher estimates in selected cohorts [6, 49, 55]. This discrepancy reflects differences in patient selection, ascertainment, background comorbidity, and expectation effects [12]. Evaluation should consider dose, interacting medicines, untreated hypothyroidism, strenuous exercise, frailty, and renal impairment before symptoms are attributed to atorvastatin [10, 11, 21]. Selected SLCO1B1 phenotypes have guideline‐supported prescribing implications, whereas other transporter markers and experimental metabolite ratios do not currently support routine decisions [56, 57]. Because symptoms also occur during placebo exposure in blinded crossover and N‐of‐1 studies, careful dechallenge and rechallenge should be used when safe rather than dismissal of the patient′s symptoms [11, 55].

### 3.2. Hepatic, Renal, and Pancreatic Toxicity

In pooled clinical trials, persistent aminotransferase elevations greater than three times the upper limit of normal occur in approximately 0.5%–0.7% of atorvastatin‐treated patients and are dose‐related; in TNT, the corresponding rates were 0.2% with 10 mg and 1.2% with 80 mg [28, 31]. Clinically apparent drug‐induced liver injury is much rarer and is supported mainly by case reports, so routine symptoms and biochemical context should be evaluated before attributing causality [13, 36]. Animal oxidative stress studies describe possible mechanisms but should not be used to estimate human risk [38, 58]. Atorvastatin requires no routine dose adjustment solely for renal clearance, although renal impairment, advanced age, and interacting drugs increase vulnerability to myopathy and rhabdomyolysis‐associated acute kidney injury [52, 59]. FAERS disproportionality signals for acute kidney injury lack denominators and are susceptible to confounding and reporting bias [52]. Acute pancreatitis is supported mainly by isolated reports and observational associations; therefore, it should be described as a possible rare event rather than an established frequent toxicity [53].

### 3.3. New‐Onset Diabetes Mellitus

New‐onset diabetes is a recognized class effect rather than a rare idiosyncratic reaction. A meta‐analysis of 13 randomized trials (91,140 participants) found a 9% relative increase in incident diabetes (odds ratio 1.09, 95% confidence interval 1.02–1.17), corresponding to approximately one additional case per 255 patients treated for four years [50]. A later individual participant meta‐analysis found relative increases of about 10% with low‐ or moderate‐intensity statin therapy and 36% with high‐intensity therapy; most excess diagnoses occurred in participants whose baseline glycemia was already close to the diagnostic threshold [51]. Absolute risk is, therefore, concentrated among patients with obesity, impaired fasting glucose, metabolic syndrome, or other diabetes risk factors. In susceptible patients, fasting plasma glucose or glycated hemoglobin should be documented before treatment and reassessed after initiation or dose intensification and periodically thereafter in accordance with diabetes care guidance; lifestyle measures should be reinforced. A modest glycemic increase alone should not prompt discontinuation when substantial cardiovascular benefit is expected [39].

### 3.4. Neurocognitive, Psychiatric, and Central Nervous System Effects

Reports of cognitive symptoms, insomnia, nightmares, depression, and peripheral neuropathy have prompted regulatory and pharmacovigilance attention, but the evidentiary strength varies [17]. Cognitive impairment and polyneuropathy are supported largely by case reports and small observational studies, and EudraVigilance disproportionality identifies reporting associations rather than causal effects [17, 18, 60]. Randomized evidence has not established a consistent increase in persistent cognitive decline [11, 15]. Proposed mechanisms from brain oxidative stress, transcriptomic, microbiome, and erythrocyte experiments remain preclinical and cannot be directly extrapolated to therapeutic use in humans [38]. Conversely, experimental reports of neuroprotection in oncology models are also preliminary [61]. Clinicians should evaluate the temporal relationship, competing neurological or psychiatric causes, dechallenge or rechallenge information, and concomitant drugs; the manuscript, therefore, uses “reported association” rather than causal language for these outcomes.

### 3.5. Hypersensitivity, Dermatologic, and Immune‐Mediated Reactions

Rare hypersensitivity, dermatologic, and immune‐mediated reactions are supported mainly by case reports that extend beyond conventional muscle and liver toxicities [62]. Reported presentations include angioedema, urticaria, eczematous rashes, thrombocytopenia, and lupus‐ or dermatomyositis‐like syndromes [19, 20, 63, 64]. Because incidence and causality cannot be established from isolated reports, clinicians should assess timing, alternative causes, concomitant medicines, and the response to withdrawal rather than infer a general atorvastatin risk. Severe persistent or systemic abnormalities following atorvastatin administration warrant prompt evaluation [13].

### 3.6. Other Reported and Experimental Organ‐Specific Effects

Ophthalmologic, neurologic, reproductive, and other organ‐specific findings are uncommon and are supported mainly by case reports, observational analyses, or preclinical experiments [65–67]. Diplopia, ptosis, peripheral neuropathy, pancreatitis, and psychiatric events should, therefore, be regarded as possible signals rather than quantified causal risks [23, 53, 68]. Suggested reproductive or oncology‐related benefits and harms remain experimental and hypothesis‐generating [61, 66, 67]. These findings warrant targeted evaluation when symptoms arise but do not justify routine discontinuation in asymptomatic patients. Decisions should remain anchored to expected cardiovascular benefit, temporal plausibility, alternative causes, and response to supervised dechallenge or rechallenge. Figure 2 separates established clinical manifestations from reported neurologic signals and labels the corresponding neurologic mechanisms as proposed and clinically unconfirmed.

**Figure 2:**
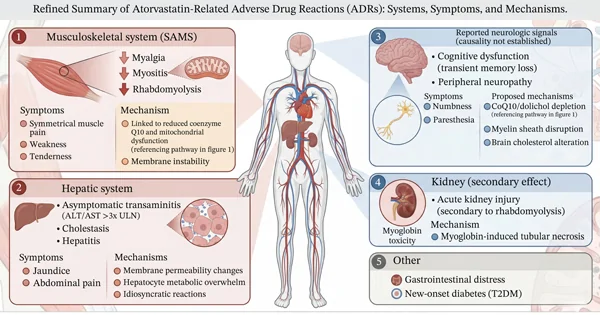
Evidence‐stratified summary of atorvastatin‐related adverse drug reactions (ADRs): systems, symptoms, and proposed mechanisms. This figure provides an evidence‐stratified overview of established adverse reactions, reported safety signals, and proposed mechanisms associated with atorvastatin. (1) Musculoskeletal system (SAMS): It covers a spectrum from symmetrical myalgia and weakness to rare, life‐threatening rhabdomyolysis; mechanisms involve mitochondrial dysfunction, CoQ10 depletion, and muscle cell membrane instability. (2) Hepatic system: It includes asymptomatic transaminitis (ALT/AST > 3x ULN), cholestasis, and hepatitis; mechanisms are linked to changes in hepatocyte membrane permeability and metabolic overwhelm. (3) Reported neurologic signals: Cognitive complaints and peripheral neuropathy have been reported, but causal relationships with atorvastatin remain unestablished. The depicted pathways involving CoQ10/dolichol depletion, myelin, and brain cholesterol are proposed mechanisms and should be interpreted as hypothesis‐generating. (4) Kidney (secondary effect): It is primarily acute kidney injury secondary to rhabdomyolysis, driven by myoglobin‐induced tubular necrosis. (5) Other: It includes gastrointestinal distress and the clinically significant risk of new‐onset Type 2 diabetes mellitus (T2DM).

## 4. Mechanistic, Pharmacogenomic, and Interaction Studies of Atorvastatin

Atorvastatin safety is influenced by CYP3A4 metabolism, glucuronidation, and hepatic transport [33]. OATP1B1 (SLCO1B1) mediates hepatic uptake, whereas ABCB1 and ABCG2 contribute to efflux and exposure variability [5, 21, 42]. However, clinically actionable atorvastatin pharmacogenomic evidence is currently limited to selected SLCO1B1 phenotypes. ABCG2 and ABCB1 findings remain exploratory for atorvastatin, whereas UGT1A1 has no validated or guideline‐supported role in routine atorvastatin dose selection; none should be treated as equivalent to SLCO1B1 [26, 42, 46, 56, 69]. The clinically relevant priority is, therefore, to identify interacting drugs, high doses, hepatic dysfunction, frailty, and prior SAMS before considering exploratory biomarkers or formulations.

### 4.1. Pharmacokinetics, Transporters, and Drug–Drug Interactions

Atorvastatin is a CYP3A4 substrate and depends on hepatic uptake and efflux transporters; active hydroxy metabolites and lactone formation also contribute to exposure [25, 26, 70, 71]. Strong CYP3A4 inhibitors, cyclosporine, some antivirals, and interacting lipid‐lowering drugs can increase myopathy risk, whereas enzyme inducers may reduce atorvastatin exposure [21]. Sacubitril/valsartan‐associated rhabdomyolysis is documented mainly in case reports and should not be generalized as a common class interaction [23]. Before dose escalation, clinicians should review prescriptions, over‐the‐counter products, grapefruit intake, hepatic status, renal vulnerability, and prior muscle symptoms. Experimental metabolite ratios and microbiome findings are promising research tools but are not validated routine tests. Table 3 categorizes the principal determinants of atorvastatin‐related adverse reactions as modifiable, nonmodifiable, or evidence‐limited risk factors and links them to corresponding clinical actions.

**Table 3:** Key risk factors influencing atorvastatin‐related adverse drug reactions (ADRs).

Risk type	Factor	Effect on ADR risk	Mechanism/rationale	Clinical action
Modifiable	Higher dose, especially 40–80 mg [15, 34, 41, 59, 72]	Dose‐related increase in SAMS and aminotransferase elevation	Greater systemic and tissue exposure	Use the lowest dose that achieves the required LDL‐C reduction; consider combination therapy
	CYP3A4/OATP inhibitors and interacting drugs [3, 5, 21–23]	Can markedly increase myopathy risk	Reduced metabolism or hepatic uptake	Reconcile medicines and avoid, limit dose, or select an alternative statin
	Untreated hypothyroidism, heavy exertion, and alcohol excess [73–75]	May mimic or amplify muscle or liver symptoms	Competing or additive injury	Correct reversible factors before declaring intolerance
	Poor glycemic profile/obesity [50, 51]	Raises absolute risk of new‐onset diabetes	Baseline glucose near diagnostic threshold	Lifestyle support and guideline‐based glucose monitoring

Nonmodifiable	Age ≥ 65 years, frailty, and low muscle mass [74, 76]	Higher susceptibility to muscle and renal complications	Reduced physiological reserve and polypharmacy	Start conservatively and monitor symptoms/interactions
	Renal or hepatic impairment [74, 75]	Raises vulnerability to toxicity and complications	Altered exposure or reduced reserve	Assess organ function and apply label‐based precautions
	SLCO1B1 rs4149056 reduced/poor function [25, 42, 57, 69]	Higher atorvastatin exposure and possible SAMS risk	Reduced OATP1B1‐mediated hepatic uptake	Apply CPIC/DPWG guidance when genotype is available or testing is clinically justified

Evidence‐limited	ABCG2 rs2231142 and ABCB1 variants [24, 26, 42, 56, 69]; UGT1A1 has no current guideline‐supported atorvastatin dosing role [42, 46, 56, 69]	Pharmacokinetic or association signals for ABCG2/ABCB1; no validated dosing role for these markers	Altered efflux transport: Efflux transport may influence exposure; any UGT1A1 contribution remains unestablished	Do not use for routine atorvastatin dosing; evidence remains exploratory

### 4.2. Pharmacogenomics and Personalized Risk of Adverse Effects

Clinically actionable evidence is currently concentrated on SLCO1B1. The reduced function c.521 T > C variant (rs4149056; included in SLCO1B1∗5, ∗15, and ∗17 haplotypes) decreases hepatic uptake and can increase atorvastatin exposure and SAMS risk [24, 26]. CPIC recommends an atorvastatin starting dose no higher than 40 mg/day for decreased or possibly decreased SLCO1B1 function and no higher than 20 mg/day for poor function, with disease‐specific titration and consideration of nonstatin combination therapy when greater LDL‐C lowering is required [42]. DPWG considers pre‐emptive testing potentially beneficial when additional myopathy risk factors are present and advises an alternative statin or the lowest effective atorvastatin dose for c.521 T > C carriers [69]. By contrast, ABCG2 c.421C > A (rs2231142) may alter atorvastatin pharmacokinetics, but CPIC provides no atorvastatin dosing recommendation; ABCB1 associations remain insufficiently validated, and UGT1A1 has no current guideline‐supported role in atorvastatin prescribing [26, 42]. These markers should not guide routine dosing [26, 42, 56, 69]. PharmGKB annotations summarize available guidance but do not constitute an independent prescribing recommendation [77].

### 4.3. Experimental Tissue‐Level Mechanisms and Delivery Platforms

Preclinical animal and in vitro studies have reported oxidative stress, mitochondrial or membrane effects, altered spermatogenesis, microbiome changes, and other tissue‐specific responses at heterogeneous doses and exposures [53, 67, 77, 78]. These findings are hypothesis‐generating because species differences, nontherapeutic concentrations, small samples, and surrogate endpoints limit clinical translation. Nanocrystals, niosomes, cocrystals, and hydrogels remain investigational delivery platforms; none has established superior long‐term safety or cardiovascular outcomes in routine atorvastatin therapy [79–81].

## 5. Clinical Management of Atorvastatin Side Effects and Risk–Benefit Perception

Management should begin with confirmation of the indication and expected absolute cardiovascular benefit, followed by review of symptom timing, creatine kinase when clinically indicated, thyroid status, hepatic function, interacting drugs, and alternative causes [2, 39]. For suspected SAMS, temporary interruption followed by lower dose rechallenge, alternate dosing, or a different statin can help establish tolerability; ezetimibe or other guideline‐directed nonstatin therapy can preserve LDL‐C lowering when the tolerated statin dose is insufficient [82, 39]. Blinded N‐of‐1 or crossover approaches can be useful for selected patients with persistent subjective symptoms, but they complement rather than replace clinical assessment [11]. Pharmacogenomic testing is most relevant when SLCO1B1 results are already available or when additional myopathy risks make testing potentially useful [24]. Experimental formulations and protective co‐treatments should not be described as established clinical strategies.

### 5.1. Randomized and Observational Trials on Side Effect Perception and Adherence

Randomized crossover trials help separate pharmacological symptoms from expectation effects. In SAMSON, symptom intensity was similar during statin and placebo periods, producing a nocebo ratio of approximately 0.90; nevertheless, the trial does not imply that all symptoms are unreal or that true SAMS never occurs [10, 83]. Observational surveys report higher symptom and discontinuation rates than blinded trials because they include broader populations but are also vulnerable to expectation, selection, and attribution biases [49]. A patient‐centered approach should validate symptoms, assess temporal and biochemical evidence, identify interacting drugs and alternative causes, and use supervised withdrawal and rechallenge when safe [10, 55]. Explaining individualized results can facilitate resumption of lipid‐lowering therapy without dismissing genuine adverse effects [84].

### 5.2. Population Pharmacovigilance, Coding, and Signal Detection

FAERS and EudraVigilance are spontaneous reporting systems designed for signal detection, not incidence estimation or causal inference [18, 36]. Disproportionality metrics such as the reporting odds ratio, proportional reporting ratio, Bayesian confidence propagation neural network, and empirical Bayes geometric mean compare reporting patterns but do not provide population risk because exposure denominators are absent [18, 52]. Results are affected by underreporting, stimulated and notoriety reporting, duplicate or incomplete reports, confounding by indication and comorbidity, differential market exposure, and uncertain diagnostic verification. A signal for psychiatric, renal, metabolic, or other events should, therefore, be described as a reporting association that requires confirmation in controlled pharmacoepidemiologic studies [18, 36]. These limitations apply whenever FAERS or EudraVigilance findings are cited in this review.

### 5.3. Computational Detection and Quantification of Atorvastatin ADR Signals

Electronic health record and claims analyses can add denominators, temporal information, and comparator groups that spontaneous reporting systems lack [36, 85]. Machine learning feature selection may screen many candidate outcomes, but it can amplify coding errors, surveillance bias, time‐varying confounding, and multiple testing problems [36]. Signals should, therefore, be validated in an independent dataset with a prespecified outcome definition, active comparator where possible, transparent confounder adjustment, and clinically interpretable effect estimates. Computational detection is best viewed as a prioritization step rather than confirmation that atorvastatin caused an adverse reaction [85].

### 5.4. Investigational Mitigation Strategies and Protective Co‐Treatments

Established mitigation strategies include dose adjustment, interaction avoidance, supervised rechallenge, use of a different statin, and addition of guideline‐directed nonstatin therapy. Nutraceutical co‐treatments and novel formulations, including cocrystals, nanocrystals, niosomes, and hydrogels, remain investigational; current evidence does not establish fewer patient‐important adverse events or improved cardiovascular outcomes [58, 79–81, 86, 87]. These should, therefore, not be used as current routine protection against atorvastatin toxicity. Figure 3 summarizes the clinically supported management pathway according to symptom severity, laboratory abnormalities, reversible risk factors, and expected cardiovascular benefit.

**Figure 3:**
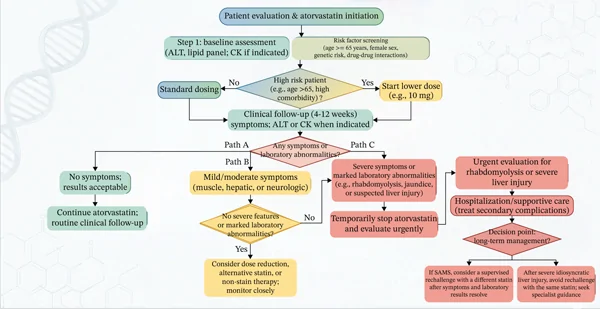
Proposed refined safety management strategy for atorvastatin therapy: a clinical decision flowchart. This clinical decision flowchart presents a proposed practical framework for individualized atorvastatin safety management. Initial assessment: Before treatment, obtain a lipid panel and baseline alanine aminotransferase; measure creatine kinase when muscle symptoms or predisposing risk factors make it clinically indicated. Screen for age, frailty, comorbidity, genetic risk when known, and drug–drug interactions. Individualized initiation: Patients with greater susceptibility may warrant a lower starting dose and earlier clinical follow‐up. At approximately 4–12 weeks, reassess response and symptoms; repeat alanine aminotransferase or creatine kinase when clinically indicated. ADR management: Path A (no symptoms and acceptable findings) supports continued therapy with routine clinical follow‐up. Path B (mild or moderate muscle, hepatic, or neurologic symptoms without severe features or marked laboratory abnormalities) supports individualized dose reduction, an alternative statin, nonstatin therapy, and close monitoring. Path C (severe symptoms or marked laboratory abnormalities, e.g., suspected rhabdomyolysis or severe liver injury) requires temporary discontinuation and urgent evaluation. Long‐term strategy: After SAMS and relevant laboratory abnormalities resolve, a supervised rechallenge with a different statin may be considered. After severe idiosyncratic liver injury, avoid rechallenge with the same statin and seek specialist guidance before considering any alternative lipid‐lowering strategy.

## 6. Summary

Atorvastatin has a favorable overall benefit–risk profile supported by randomized trials and meta‐analyses [1, 4, 27]. Serious adverse reactions are uncommon, but tolerability concerns are clinically important because they can reduce adherence [2, 32, 33, 35]. The most actionable safety determinants are dose, interacting drugs, hepatic status, frailty, renal vulnerability to rhabdomyolysis, prior SAMS, and selected SLCO1B1 phenotypes [24]. New‐onset diabetes is a recognized small risk rather than a rare idiosyncratic event: Relative risk increases by roughly 9%–12%, with the greatest absolute excess among patients already near the diagnostic threshold, whereas cardiovascular benefit generally remains larger [27, 50].

Evidence hierarchy is essential when counseling patients. Randomized and synthesized human evidence should drive estimates of benefit and common harm, observational studies extend generalizability, spontaneous reports and case reports identify signals, and animal or in vitro experiments explain possible mechanisms without proving clinical toxicity. This distinction is especially important for neuropsychiatric, reproductive, microbiome, renal, pancreatic, and novel formulation claims, many of which remain uncertain. Pharmacovigilance signals must be interpreted in light of underreporting, duplicate reports, missing denominators, confounding, and inability to establish causality.

Current priorities are to standardize SAMS phenotyping, validate biomarkers across diverse populations, test whether genotype‐guided prescribing improves patient‐important outcomes, and quantify absolute adverse event risks with active comparator studies. Clinically, the most effective strategy is shared decision‐making that communicates absolute cardiovascular benefit, acknowledges symptoms, corrects reversible risk factors, and maintains the maximally tolerated lipid‐lowering regimen through dose adjustment, rechallenge, statin substitution, or nonstatin combination therapy. Experimental delivery systems should advance only after demonstrating reproducible pharmacokinetics, safety, and cardiovascular outcomes in humans.

## Author Contributions

Hanyu Wang: conceptualization, methodology, software, investigation, and writing—original draft. Yue Pan: methodology, validation, formal analysis, investigation, and writing—original draft. Yiming Li: data curation, visualization, and resources. Pan Zhai: conceptualization, supervision, project administration, funding acquisition, and writing—review and editing. Hanyu Wang and Yue Pan contributed equally to this study.

## Funding

This work was supported by the Hubei Provincial Natural Science Foundation (Grant No. 2025AFC076), the Hubei Provincial Administration of Traditional Chinese Medicine Scientific Research Project ‐ Traditional Chinese Medicine Joint Fund (Grant No. ZY2025L135), and the Science and Technology Innovation Cultivation Fund Project of Zhongnan Hospital of Wuhan University (Grant No. CXPY202567).

## Disclosure

All authors have read and approved the submitted version of the manuscript.

## Conflicts of Interest

The authors declare no conflicts of interest.

## Data Availability

Data sharing is not applicable to this article, as no datasets were generated or analyzed during the current study.
